# Mutant IDH1 expression is associated with down-regulation of monocarboxylate transporters

**DOI:** 10.18632/oncotarget.9006

**Published:** 2016-04-26

**Authors:** Pavithra Viswanath, Chloe Najac, Jose L. Izquierdo, Aleksandr Pankov, Chibo Hong, Pia Eriksson, Joseph F. Costello, Russell O. Pieper, Sabrina M. Ronen

**Affiliations:** ^1^ Department of Radiology and Biomedical Imaging, University of California San Francisco, San Francisco, CA 94143, USA; ^2^ Department of Neurological Surgery, Helen Diller Research Center, University of California San Francisco, San Francisco, CA 94143, USA

**Keywords:** MCT1, MCT4, mutant IDH1, metabolic reprogramming, magnetic resonance spectroscopy

## Abstract

Mutations in isocitrate dehydrogenase 1 (IDH1) are characteristic of low-grade gliomas. We recently showed that mutant IDH1 cells reprogram cellular metabolism by down-regulating pyruvate dehydrogenase (PDH) activity. Reduced pyruvate metabolism via PDH could lead to increased pyruvate conversion to lactate. The goal of this study was therefore to investigate the impact of the IDH1 mutation on the pyruvate-to-lactate flux. We used ^13^C magnetic resonance spectroscopy and compared the conversion of hyperpolarized [1-^13^C]-pyruvate to [1-^13^C]-lactate in immortalized normal human astrocytes expressing mutant or wild-type IDH1 (NHAIDHmut and NHAIDHwt). Our results indicate that hyperpolarized lactate production is reduced in NHAIDHmut cells compared to NHAIDHwt. This reduction was associated with lower expression of the monocarboxylate transporters MCT1 and MCT4 in NHAIDHmut cells. Furthermore, hyperpolarized lactate production was comparable in lysates of NHAIDHmut and NHAIDHwt cells, wherein MCTs do not impact hyperpolarized pyruvate delivery and lactate production. Collectively, our findings indicated that lower MCT expression was a key contributor to lower hyperpolarized lactate production in NHAIDHmut cells. The SLC16A3 (MCT4) promoter but not SLC16A1 (MCT1) promoter was hypermethylated in NHAIDHmut cells, pointing to possibly different mechanisms mediating reduced MCT expression. Finally analysis of low-grade glioma patient biopsy data from The Cancer Genome Atlas revealed that MCT1 and MCT4 expression was significantly reduced in mutant IDH1 tumors compared to wild-type. Taken together, our study shows that reduced MCT expression is part of the metabolic reprogramming of mutant IDH1 gliomas. This finding could impact treatment and has important implications for metabolic imaging of mutant IDH1 gliomas.

## INTRODUCTION

Metabolic reprogramming is an essential hallmark of cancer [1, 2]. Studies indicate that the oncogenes and tumor suppressor genes that initiate and maintain tumorigenesis also reprogram metabolism to cater to the biosynthetic demands associated with oncogenic transformation [3-6]. A common feature of many types of cancer cells is the phenomenon of aerobic glycolysis, also known as the Warburg effect [7, 8]. In the presence of oxygen, normal differentiated cells metabolize glucose to pyruvate through glycolysis followed by oxidation of pyruvate via the tricarboxylic acid (TCA) cycle. Cancer cells, on the other hand, metabolize glucose mostly to lactate, even under aerobic conditions [9]. At the molecular level, the Warburg effect is driven, amongst other factors, by overexpression of lactate dehydrogenase A (LDHA), the enzyme mediating the NADH-dependent reduction of pyruvate to lactate [7]. To avoid the associated reduction in intracellular pH and resultant cellular death, cancer cells also up-regulate expression of several transporters including the monocarboxylate transporters 1 and 4 (MCT1 and MCT4) [10-13].

The recent discovery that mutations in metabolic enzymes such as succinate dehydrogenase, fumarate hydratase and isocitrate dehydrogenase (IDH) can facilitate malignancy further emphasizes the connection between altered metabolism and cancer [4]. Most notably, the cytosolic isoform of IDH (IDH1), which is mutated in 70-90% of low-grade gliomas and secondary glioblastomas, is now understood to function as a “driver” of tumorigenesis [14-16]. The wild-type IDH1 enzyme converts isocitrate to α-ketoglutarate (α-KG). In contrast, mutations in the enzyme lead to its neomorphic ability to convert α-KG to 2-hydroxyglutarate (2-HG) [17, 18]. 2-HG is a competitive inhibitor of several α-KG-dependent dioxygenases including the TET family of DNA hydroxylases, the Jumonji C family of histone demethylases, and prolyl hydroxylases [19]. The resulting hypermethylator phenotype is thought to lead to a block in differentiation and the initiation of tumorigenesis in mutant IDH1 cells [19-21].

Several studies have demonstrated that mutant IDH1 cells also undergo extensive metabolic reprogramming that extends beyond 2-HG production. Reitman et al. were the first to perform a metabolomic analysis of wild-type and mutant IDH1 glioma cells. They showed that levels of several amino acids and TCA cycle intermediates were reduced in mutant IDH1 cells relative to wild-type [22] and similar results were obtained in a subsequent study of patient-derived mutant IDH1 tumor samples [23]. Reitman et al. also found reduced levels of phosphocholine (PC) and increased levels of glycerophosphocholine (GPC) in mutant IDH1 glioma cells relative to wild-type cells [22] and these results were in line with a subsequent study by Esmaeili et al. that showed increased GPC levels in mutant IDH1 gliomas relative to wild-type tumors [24]. These findings run counter to the increase in PC and drop in GPC typically observed in cancer [25] and suggest that mutant IDH1 cells reprogram their metabolism differently. Another indicator of unusual metabolic reprogramming in mutant IDH1 gliomas is their silencing of LDHA expression shown by Chesnelong et al. to occur via promoter hypermethylation in patient-derived mutant IDH1 glioma models and patient samples [26].

Magnetic resonance spectroscopy (MRS) is a translational method that can be used to monitor metabolism in cell extracts, live cells, tissue biopsies, animals and patients *in vivo* [27, 28]. Steady-state metabolite concentrations are primarily monitored using ^1^H and ^31^P MRS and metabolic fluxes can be assessed using ^13^C MRS to determine the fate of ^13^C-labeled metabolites. The recent development of dissolution dynamic nuclear polarization (DNP) for ^13^C-labeled metabolites enables a signal-to-noise ratio (SNR) enhancement on the order of 10,000-50,000 allowing rapid monitoring of metabolic fluxes using ^13^C MRS [29-31]. In particular, [1-^13^C]-pyruvate is readily hyperpolarized and has been widely used to probe the Warburg effect in cancer cells, animals, and recently in prostate cancer patients [31-33].

Our laboratory has used MRS to investigate the metabolic reprogramming of mutant IDH1 glioma cells. We carried out an unbiased ^1^H MRS-based metabolomic analysis of glioma cells genetically engineered to express either the wild-type or mutant IDH1 enzyme and found that steady-state levels of glutamate, lactate and PC were reduced in mutant IDH1 cells [34]. In a separate study, we linked the reduction in glutamate levels to down-regulation of pyruvate dehydrogenase (PDH) activity in mutant IDH1 cells [35]. As mentioned above, cancer cells often up-regulate pyruvate flux towards lactate while reducing pyruvate oxidation via PDH, resulting in an inverse link between PDH activity and aerobic glycolysis [36-42]. The down-regulation of PDH activity in mutant IDH1 cells might, therefore, be expected to result in increased lactate production from pyruvate in these cells. However, LDHA expression is typically silenced in mutant IDH1 gliomas [26], suggesting that pyruvate conversion to lactate could be reduced. The goal of this study was therefore to investigate the flux of pyruvate to lactate in mutant IDH1 glioma cells using hyperpolarized ^13^C MRS.

We performed our studies on an immortalized normal human astrocyte (NHA) cell line genetically engineered to express either wild-type or mutant IDH1 [34]. Our results indicate that the flux of hyperpolarized [1-^13^C]-pyruvate to hyperpolarized [1-^13^C]-lactate is significantly reduced in mutant IDH1 cells compared to wild-type and this could be linked, at least in part, to reduced MCT1 and MCT4 expression in our mutant IDH1 cells. Importantly, the drop in MCT expression was confirmed in patient data from The Cancer Genome Atlas (TCGA) database. Our findings contribute to the understanding of mutant IDH1 cell metabolism and have important implications for the treatment and imaging of mutant IDH1 gliomas.

## RESULTS

### Pyruvate to lactate flux is significantly reduced in mutant IDH1 cells

**Figure 1:**
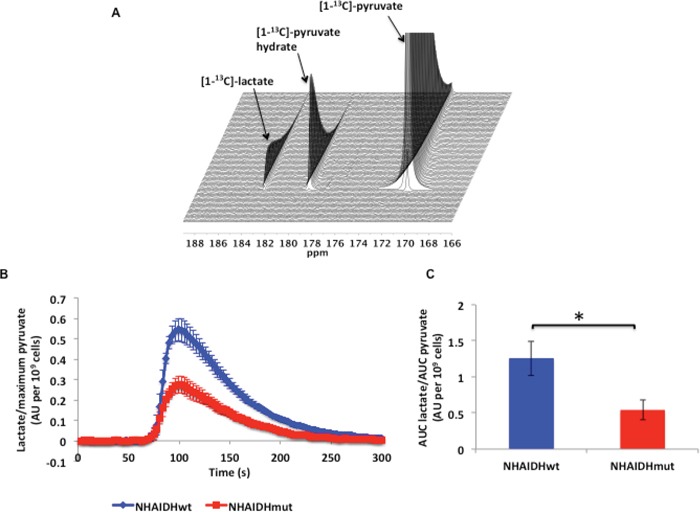
**A.** ^13^C MR spectral array showing hyperpolarized [1-^13^C]-lactate production from hyperpolarized [1-^13^C]-pyruvate in live NHAIDHwt cells. **B.** Build-up of hyperpolarized [1-^13^C]-lactate following injection of hyperpolarized [1-^13^C]-pyruvate in live NHAIDHwt and NHAIDHmut cells. **C.** Ratio of the AUC of hyperpolarized lactate to the AUC of hyperpolarized pyruvate normalized to cell number for live NHAIDHwt and NHAIDHmut cells. * represents statistically significant difference (p<0.05).

To compare pyruvate to lactate flux between NHAIDHwt and NHAIDHmut cells, we used hyperpolarized ^13^C MRS and dynamically probed the metabolism of hyperpolarized [1-^13^C]-pyruvate in live cells in a bioreactor. Following injection of hyperpolarized [1-^13^C]-pyruvate (δ=171.1 ppm) into the medium of perfused cells, production of hyperpolarized [1-^13^C]-lactate (δ=183.2 ppm) could be observed (Figure 1A). Comparison of the build-up of hyperpolarized lactate over time showed a clear reduction in hyperpolarized lactate production in NHAIDHmut cells relative to NHAIDHwt (Figure 1B). Quantification of Lac_max_/Pyr_max_ revealed that hyperpolarized [1-^13^C]-lactate production was significantly reduced by 48.6±10.5% (p<0.0005) (Figure 1B). Similarly, quantification of Lac_auc_/Pyr_auc_ showed a drop of 56.9±7.3% (p<0.005) in NHAIDHmut cells relative to NHAIDHwt cells (Figure 1C).

### LDH expression and activity, intracellular NADH concentration and NAD^+^/NADH ratio are unaltered in mutant IDH1 cells

Previous studies have demonstrated that several factors can influence the production of hyperpolarized [1-^13^C]-lactate. One factor is the expression and activity of the two LDH isoforms, LDHA and LDHB. LDHA is efficient at the NADH-dependent reduction of pyruvate to lactate while LDHB catalyzes the NAD^+^-dependent conversion of lactate to pyruvate [56]. In addition, the intracellular NADH concentration [57, 58] and the size of the intracellular lactate pool [57, 59] can modulate hyperpolarized lactate production. Finally, MCT1, which has a high affinity for the transport of pyruvate into the cell (K_m_ 0.7-3 mM) and a somewhat lower affinity for the transport of lactate (K_m_ ~3-6 mM), and MCT4, which has a low affinity for pyruvate (K_m_ ~153 mM) and a higher affinity for lactate (K_m_ ~25-30 mM), both affect hyperpolarized lactate production by controlling the delivery of pyruvate as well as the intracellular lactate pool [57, 60-63].

**Figure 2:**
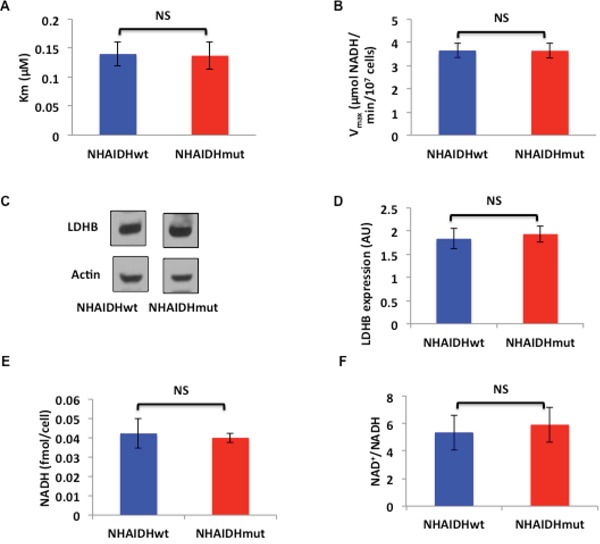
**A.** K_m_ for pyruvate of LDHA from NHAIDHwt and NHAIDHmut cells. **B.** V_max_ of LDHA from NHAIDHwt and NHAIDHmut cells. **C.** Western blots for LDHB in NHAIDHwt and NHAIDHmut cells. **D.** Quantification of LDHB expression in NHAIDHwt and NHAIDHmut cells. **E.** NADH concentration in NHAIDHwt and NHAIDHmut cells. **F.** NAD^+^/NADH ratio in NHAIDHwt and NHAIDHmut cells. NS indicates no statistically significant difference.

First, we looked at LDHA expression and activity. As mentioned earlier, LDHA expression is silenced by hypermethylation in patient-derived mutant IDH1 glioma models [26]. However, in our genetically engineered NHA model, it has been shown that the difference in LDHA expression between NHAIDHwt and NHAIDHmut cells is not statistically significant, in spite of a significant increase in promoter methylation [26]. Here we confirmed these earlier findings. We found that the LDHA gene was significantly hypermethylated in NHAIDHmut relative to NHAIDHwt (Δβ = 0.35, p<0.0001, probe cg02232751), but no significant difference in LDHA expression between NHAIDHwt and NHAIDHmut cells was observed (Supplementary Figure 1). To further confirm our results, we also measured LDHA activity and found no difference in the K_m_ (Figure 2A) and V_max_ (Figure 2B) of LDHA between NHAIDHwt and NHAIDHmut cells.

Next, we examined LDHB expression and found no significant difference between NHAIDHwt and NHAIDHmut cells (Figure 2C and 2D). Measurement of intracellular NADH concentration (Figure 2E) and the NAD^+^/NADH ratio (Figure 2F) also revealed no differences between NHAIDHwt and NHAIDHmut cells.

### Reduced glucose uptake leads to a drop in intracellular lactate production in NHAIDHmut cells and could contribute to reduced hyperpolarized [1-^13^C]-lactate production

**Figure 3:**
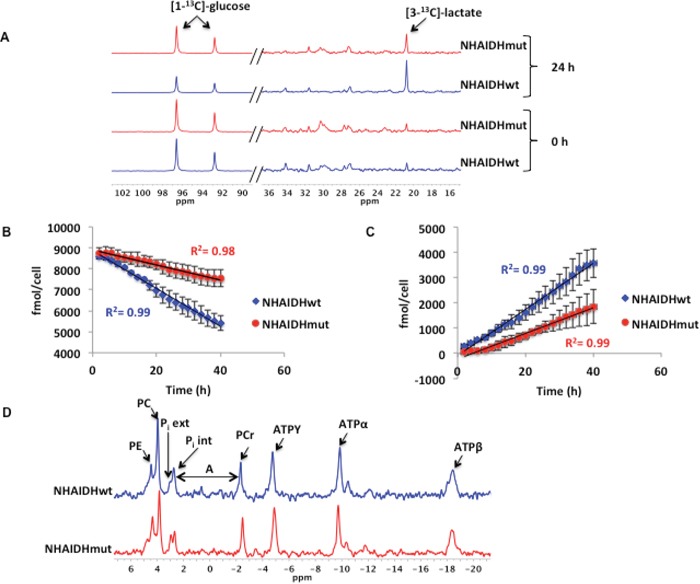
**A.** Representative spectra showing a comparison of [1-^13^C]-glucose consumption and [3-^13^C]-lactate production at 0 and 24 h in NHAIDHwt and NHAIDHmut cells (the portion of the spectrum containing [1-^13^C]-glucose (90-102 ppm) is scaled differently than that containing [3-^13^C]-lactate (16-36 ppm) in order to illustrate the difference in glucose uptake between NHAIDHwt and NHAIDHmut cells). **B.** Quantification of [1-^13^C]-glucose uptake over the course of 40 h in NHAIDHwt and NHAIDHmut cells. **C.** Quantification of [3-^13^C]-lactate production over the course of 40 h in NHAIDHwt and NHAIDHmut cells. **D.** Representative ^31^P MR spectra for NHAIDHwt and NHAIDHmut cells. The chemical shift difference (A) between the P_i_ int and PCr peaks was used to calculate the intracellular pH in NHAIDHwt and NHAIDHmut cells. PE=phosphoethanolamine, PC=phosphocholine, P_i_ ext=extracellular inorganic phosphate, P_i_ int= intracellular inorganic phosphate, PCr=phosphocreatine, ATPY=Y phosphate of ATP, ATPα= α phosphate of ATP and ATPβ= β phosphate of ATP.

Reduced intracellular lactate has been shown to lead to a drop in hyperpolarized [1-^13^C]-lactate production [57]. We previously reported that the intracellular lactate pool is lower in NHAIDHmut cells compared to NHAIDHwt (28±14.8%, p<0.05) [34]. To further examine the reasons for reduced lactate production, we metabolically labeled cells with [1-^13^C]-glucose and monitored [1-^13^C]-glucose uptake and [3-^13^C]-lactate production in NHAIDHwt and NHAIDHmut cells assembled into a bioreactor (Figure 3A). Quantification of the rate of [1-^13^C]-glucose consumption indicated that it was reduced significantly by 62.5±16.3% (p<0.05) in NHAIDHmut cells compared to NHAIDHwt cells from 90.2±17.1 fmol/cell.h to 34.9±18.5 fmol/cell.h (Figure 3B). Concomitantly, [3-^13^C]-lactate production was reduced by 45.6±13.5% (p<0.05) from 90.5±18.2 fmol/cell.h to 49±16.1 fmol/cell.h in NHAIDHmut cells relative to NHAIDHwt (Figure 3C). These results suggest that reduced lactate production from glucose is likely responsible for the lower intracellular lactate in NHAIDHmut cells. This could explain, at least in part, the observed drop in hyperpolarized [1-^13^C]-lactate production in NHAIDHmut cells.

Interestingly however, the drop in intracellular lactate levels was not associated with a detectable change in intracellular pH as assessed using ^31^P MRS (Figure 3D). The intracellular pH of NHAIDHwt cells was 7.16±0.08 and the pH of NHAIDHmut was 7.19±0.12 (p=0.8).

### Expression of MCT1 and MCT4 is down-regulated in NHAIDHmut cells

**Figure 4:**
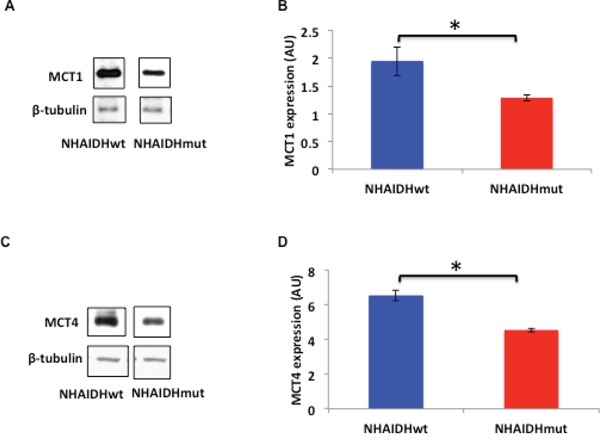
**A.** MCT1 expression in NHAIDHwt and NHAIDHmut cells determined by western blotting. **B.** Quantification of MCT1 expression in NHAIDHwt and NHAIDHmut cells. **C.** MCT4 expression in NHAIDHwt and NHAIDHmut cells determined by western blotting. **D.** Quantification of MCT4 expression in NHAIDHwt and NHAIDHmut cells. * represents statistically significant difference (p<0.05).

As mentioned earlier, MCT expression has been shown to influence hyperpolarized lactate production from pyruvate [44, 57, 63]. Therefore, we examined MCT1 and MCT4 expression in the NHA model using western blotting. Our results indicated that MCT1 expression was reduced by 33.3±6.4% (p<0.05) (Figure 4A & 4B) and MCT4 expression was reduced by 30.4±2% (p<0.005) (Figure 4C & 4D) in NHAIDHmut cells relative to NHAIDHwt.

2-HG produced by the IDH1 mutation is known to induce DNA hypermethylation resulting in reduced expression of several genes [20, 21, 26]. Examination of the methylome of NHAIDHwt and NHAIDHmut cells indicated that the SLC16A3 gene coding for MCT4 was hypermethylated in NHAIDHmut cells relative to NHAIDHwt (Δβ = 0.39, p<0.0001, probe cg09147131). However, methylation of the SLC16A1 gene coding for MCT1 was not significantly different between NHAIDHwt and NHAIDHmut cells (Δβ=0.02, p=0.1, probe cg20614262).

### Reduced MCT expression is a major factor leading to reduced hyperpolarized [1-^13^C]-lactate production in mutant IDH1 NHAs

**Figure 5:**
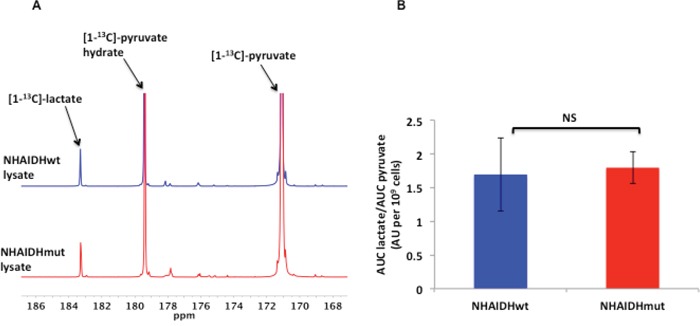
**A.** Representative ^13^C MR spectra comparing maximum hyperpolarized [1-^13^C]-lactate produced from hyperpolarized [1-^13^C]-pyruvate in NHAIDHwt and NHAIDHmut lysates. **B.** Ratio of the AUC of hyperpolarized lactate to the AUC of hyperpolarized pyruvate normalized to cell number for NHAIDHwt and NHAIDHmut lysates. NS indicates no significant difference.

The rate of pyruvate transport across the cell membrane, mediated by the MCTs and in particular by MCT1, affects the hyperpolarized pyruvate to lactate flux in live cells as previously shown in several studies [44, 57, 63]. To confirm the role of MCTs in explaining the reduction in hyperpolarized lactate production in our intact NHAIDHmut cells, we therefore measured hyperpolarized lactate production in cell lysates, wherein the role of the MCTs is excluded. Quantification of both Lac_max_/Pyr_max_ (2.5±0.5 in NHAIDHwt and 2.2±0.2 in NHAIDHmut, p=0.56) and Lac_auc_/Pyr_auc_ (1.7±0.5 in NHAIDHwt and 1.8±0.2 in NHAIDHmut, p=0.54) revealed that hyperpolarized [1-^13^C]-lactate production was comparable in NHAIDHwt lysate relative to NHAIDHmut lysate (Figure 5A and 5B). In contrast to live cells, [3-^13^C]-alanine (δ=176.2 ppm) production could be detected in lysates but showed no difference between NHAIDHwt and NHAIDHmut lysates (data not shown). These results confirmed that the reduction in hyperpolarized pyruvate to lactate flux in intact NHAIDHmut cells resulted primarily from reduced expression of the MCTs.

### MCT1 and MCT4 expression is reduced in mutant IDH1 low-grade glioma patient tumors relative to wild-type IDH1 tumors

**Figure 6:**
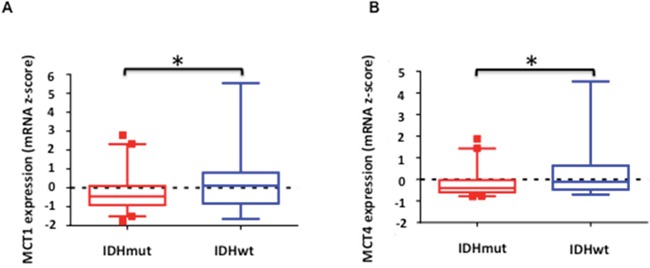
Comparison of normalized expression z-scores for MCT1 A. and MCT4 B. mRNA in low-grade glioma patient samples Boxes denote the mean z-score and whiskers denote 1-99 percentile for tumors within the group (n=68 for IDH1 wild-type and n=218 for IDH1 mutant). A negative z-score represents expression value below the population mean. * indicates statistically significant effect (p<0.05).

In order to assess whether our findings are clinically relevant, we analyzed expression data from low-grade (grade II and III) glioma biopsy samples available in the TCGA database. We found that the mean normalized z-scores of mRNA expression were significantly reduced for both MCT1 (Figure 6A, 0.22 vs. −0.21, p<0.05) and MCT4 (Figure 6B, 0.25 vs. −0.15, p<0.005) in mutant IDH1 glioma samples (n=218) compared to wild-type IDH1 low-grade glioma (n=68). This confirmed that our findings in the NHA model reflected the behavior of mutant IDH1 tumors in patients.

## DISCUSSION

Using a combination of MRS, cell and molecular biological assays, our study found that the expression of the monocarboxylate transporters MCT1 and MCT4 was reduced in our genetically engineered mutant IDH1 cells when compared to their wild-type IDH1 counterparts.

Whereas recently isolated patient-derived mutant IDH1 models [26] are clinically more relevant, they do not provide a matched comparison between IDH1 wild-type and mutant cells. In the current study we therefore used immortalized NHAs genetically engineered to express either the wild-type or mutant IDH1 enzyme in order to obtain a paired comparison of the effect of the IDH1 mutation on MCT expression. A limitation of our model is that the NHAIDHwt cells are not tumorigenic and therefore an *in vivo* comparison is not possible. Furthermore, the NHAIDHmut cells have not silenced LDHA [26] and therefore do not fully reproduce the findings from patient tumors. This could be due to their relatively low passage number (~P20) following mutant IDH1 transfection, considering that remodeling of the methylome has been shown to occur progressively [20]. Importantly, however, our observation that the expression of MCT1 and MCT4 is reduced in NHAIDHmut cells was validated in patient samples submitted to the TCGA. This confirms the clinical significance of our findings with regard to the MCTs.

By comparing hyperpolarized pyruvate to lactate conversion in lysates and in intact cells, we have identified the role of down-regulated MCT1 and MCT4 expression in reducing hyperpolarized lactate production in mutant IDH1 cells. However, it should be noted that lysing cells not only removes the cell membrane (and therefore the MCTs), but also dilutes the intracellular lactate. We have previously shown that intracellular lactate levels are lower by 28±14.8% (p<0.05) in mutant IDH1 cells [34] and others have demonstrated that a reduction in intracellular lactate levels can reduce hyperpolarized lactate production [57]. We therefore cannot rule out that lower intracellular lactate levels in mutant IDH1 cells also play a role in reduced hyperpolarized lactate production.

With regard to the relative contribution of MCT1 and MCT4 towards hyperpolarized pyruvate transport, based on the substrate affinities mentioned above, MCT1 is generally thought to mediate pyruvate uptake into the cells and MCT1 and MCT4 to mediate lactate efflux. However, transport also depends on the concentration gradients of monocarboxylate and H^+^ [62, 64]. Thus, MCT1 and MCT4 could both serve to import as well as export pyruvate into and out of the cell, albeit with differing affinities. Further functional studies with MCT1/4 inhibitors and genetic knockdowns are needed to unravel the relative contributions of MCT1 and MCT4 towards hyperpolarized pyruvate transport in mutant IDH1 gliomas.

Interestingly, our data indicates that the mechanisms through which MCT1 and MCT4 are silenced could be different. Reduced MCT4 expression in NHAIDHmut cells was associated with increased promoter methylation of the SLC16A3 (MCT4) gene, which can explain the drop in MCT4 expression [65]. Although our results were based on two replicates for each cell line, they are in agreement with those of Turcan et al. who, using NHAs of similar origin as our study, found that the SLC16A3 (MCT4) gene was hypermethylated in mutant IDH1 cells (22.9 fold, p<0.05) relative to wild-type cells [20]. Our findings are also in agreement with a previous study showing that SLC16A3 (MCT4) mRNA levels were lower in patient-derived mutant IDH1 models compared to wild-type IDH1 glioblastoma models [26]. Most importantly, our results are in line with the TCGA data wherein a reduction in SLC16A3 (MCT4) mRNA levels was observed in mutant IDH1 patient biopsy samples relative to wild-type. Taken together, these findings suggest that MCT4 expression is likely down-regulated in mutant IDH1 cells via promoter hypermethylation. In contrast, SLC16A1 (MCT1) methylation was comparable in our NHAIDHwt and NHAIDHmut cells pointing to a possible alternate mechanism for reduced expression of this gene. In this case, our findings differ from those of Turcan et al. who found that the SLC16A1 (MCT1) gene is significantly methylated (1.7 fold, p<0.05) in mutant IDH1 cells compared to wild-type [20]. However, it should be noted that the cells used in that study were at a higher passage number (P40), possibly indicating that, ultimately, mutant IDH1 cells reduce MCT1 expression via more than one mechanism. Indeed, a recent paper identified methylation-independent modulation of gene expression via insulator function modification in mutant IDH1 cells [66]. Further studies are needed to fully understand the mechanisms by which MCT1 and MCT4 expression and function are modulated in mutant IDH1 cells.

Independent of mechanism, our findings highlight another aspect of the unusual metabolic reprogramming of mutant IDH1 cancer cells. Previous studies have shown, for example, that abnormal choline metabolism with increased PC synthesis is a hallmark of many types of cancer cells [25]. Mutant IDH1 glioma cells, however, display reduced PC levels indicating that they reprogram choline metabolism differently [34]. Likewise, most types of cancer cells display the Warburg effect with elevated glucose uptake and conversation to lactate mediated, amongst other factors, by overexpression of LDHA [7, 8]. Our results indicate that mutant IDH1 cells, on the other hand, reduce glucose uptake and concomitant lactate production, in addition to silencing LDHA expression [26], making their metabolic phenotype different from non-IDH mutated cancer cells. With regard to MCT1 and MCT4, previous studies have shown that cancer cells up-regulate these transporters in order to facilitate lactate export and avoid intracellular acidosis [7, 10-13]. Our finding that expression of MCTs is reduced in mutant IDH1 gliomas is therefore also unusual. However, it is in line with a study showing that NHE-1, another transporter involved in maintenance of intracellular pH and cell survival in glycolytic tumors, is silenced in 1p/19q co-deleted mutant IDH1 gliomas [67, 68]. Silencing of NHE-1 and reduced MCT expression are consistent with reduced glycolytic production of lactate as well as silencing of LDHA expression [26]; cells that produce less lactate do not require an increase in its export and are, therefore, less dependent on MCT and NHE-1 expression.

Reduced MCT1 and MCT4 expression in mutant IDH1 gliomas could also have important implications for chemotherapy. Elevated expression of the MCTs has been observed in highly glycolytic tumors and has been associated with poor outcome [11, 69, 70]. Additionally, the MCTs have been shown to impact the effects of chemotherapeutic agents such as 3-bromopyruvate and dichloroacetate by modulating their delivery to the cell [71-73]. The MCTs can also sensitize cells to radiation therapy [74, 75]. As a result, MCTs have been considered a therapeutic target, either alone or in combination with other therapies [61, 69, 70, 74, 76-79]. However, in the current study, we show that expression of MCT1 and MCT4 is significantly reduced in mutant IDH1 gliomas. Further studies are needed to assess the utility of MCT inhibition as a treatment strategy for mutant IDH1 gliomas, but considering that the effectiveness of MCT inhibitors has been linked to MCT expression [78], inhibition of MCT expression and/or function likely would not provide a therapeutic opportunity for mutant IDH1 gliomas and this point should be considered in the planning of treatment for mutant IDH1 glioma patients. Nonetheless other metabolic targets such as mutant IDH and PDH may provide potential therapeutic opportunities [35, 80].

Hyperpolarized ^13^C MR imaging is rapidly emerging as a novel imaging modality in oncology, and its safety and utility in prostate cancer patients has been demonstrated [31-33]. Positron emission tomography imaging using ^18^F-fluorodeoxyglucose is of limited utility in brain tumors due to the high background glucose consumption in normal brain, which limits sensitivity and specificity [81]. Hyperpolarized [1-^13^C]-pyruvate has been shown to cross the blood-brain barrier in non-human primates [82] and initial clinical studies also confirm that this is the case in patients. Furthermore, detection of increased hyperpolarized [1-^13^C]-lactate production from hyperpolarized [1-^13^C]-pyruvate as a result of up-regulation of aerobic glycolysis in high-grade glioblastomas and a drop in hyperpolarized [1-^13^C]-lactate in response to treatment have been reported in animal models [33, 49, 83, 84]. Hyperpolarized ^13^C MRS of pyruvate therefore has clear potential as an imaging method for brain tumors. However, as mentioned earlier, production of hyperpolarized lactate depends on pyruvate transport into the cell via the MCTs, LDHA expression, intracellular lactate levels and NADH. Our finding regarding reduced expression of the MCTs, taken together with reduced intracellular lactate levels and the silencing of LDHA expression [26], indicate that in mutant IDH1 gliomas *in vivo* the production of hyperpolarized lactate is likely to be very limited. While a head-to-head comparison of NHAIDHwt and NHAIDHmut tumors is not possible *in vivo* due to the lack of tumorigenicity of the NHAIDHwt cells, a comparison of patient-derived mutant IDH1 models, such as BT142, with wild-type IDH1 glioblastoma models, such as the U87 model, is feasible and provides a clinically relevant comparison. Such a preliminary study from our lab indicated that hyperpolarized pyruvate to lactate conversion was very significantly reduced in the BT142 model compared to the U87 model [85]. While this could preclude the use of hyperpolarized lactate production as an indicator of response to therapy it could provide a method of confirming mutant IDH1 status [84].

In summary, our finding that the expression of MCT1 and MCT4 is reduced in mutant IDH1 gliomas highlights the unusual metabolic reprogramming that occurs in mutant IDH1 tumors and has important implications for our understanding of these tumors and their treatment. Furthermore, and importantly, our findings in preclinical models point to what is likely an unusual fate for hyperpolarized pyruvate and an unexpected metabolic imaging signature for these tumors in the clinic.

## MATERIALS AND METHODS

### Cell culture

Normal human astrocytes (Clonetics) were immortalized as described previously [43] and transfected with lentiviral vectors expressing the wild-type IDH1 gene (NHAIDHwt) or mutant IDH1 R132H gene (NHAIDHmut) [34]. There was no significant difference in doubling time between NHAIDHwt and NHAIDHmut cells [34]. Cell lines were last authenticated by single nucleotide polymorphism fingerprinting (Cell Line Genetics) on 10/28/2015. Cells were routinely cultured in Dulbecco's Modified Eagles' Medium (UCSF Cell Culture Facility, UCSF) supplemented with 10% fetal bovine serum (Corning), 2 mM L-glutamine (UCSF Cell Culture Facility, UCSF) and 100 U/ml each of penicillin and streptomycin (UCSF Cell Culture Facility, UCSF).

### MRS studies

MRS studies were performed on live cells immobilized on Biosilon polystyrene beads (Nunc) using an MR-compatible cell perfusion (bioreactor) system as described previously [44]. All MRS studies were performed on a 500 MHz INOVA spectrometer (Agilent) equipped with a 10 mm broadband probe. First, to confirm cell viability, proton-decoupled ^31^P spectra were acquired using a 30° pulse, 3 s relaxation delay and 1024 scans. For thermally polarized ^13^C MRS studies, glucose in the medium was replaced with 5 mM [1-^13^C]-glucose (Sigma-Aldrich). Proton-decoupled ^13^C spectra were then acquired in 2 h blocks using a 60° pulse, 6 s relaxation delay and 2400 scans over the course of 40 h. Peak integrals were quantified using Mnova (Mestrelab Research), corrected for saturation and NOE and normalized to cell number and to the known concentration of [1-^13^C]-glucose at the start of the experiment. The rates of [1-^13^C]-glucose uptake and [3-^13^C]-lactate production were then detemined using linear regression analysis. For hyperpolarized ^13^C MRS, [1-^13^C]-pyruvic acid (Sigma Aldrich) containing 15 mM of the OX063 trityl radical (Oxford Instruments) was hyperpolarized using the Hypersense DNP polarizer (Oxford Instruments) as described previously [44]. After ~1 h, hyperpolarized pyruvic acid was rapidly dissolved in 6 mL isotonic buffer (40 mM Tris HCl, 3 μM ethylenediaminetetraacetic acid (EDTA), pH 7.8) and injected into the perfusion system within 15 s at approximately 37°C and to a final concentration of 5 mM. For studies of cell lysates, cells (~10^8^) were lysed by sonication in 80 mM Tris HCl, 200 mM NaCl, 2 mM NADH, pH 8, centrifuged at 14000 rpm for 10 minutes to remove precipitated membranes and the supernatant placed in a 10 mM NMR tube. Hyperpolarized pyruvate was then injected to a final concentration of 5 mM. Single transient ^13^C spectra were acquired every 3 s over a period of 300 s using 5° pulses, 40 k data points and a spectral width of 20 kHz. Data analysis was performed using Mnova. Several approaches have been described to analyze hyperpolarized pyruvate metabolism and results are essentially comparable [45-49]. Here, the data was assessed using two model-free approaches. Either the integral of lactate was normalized to the maximum pyruvate integral and to cell number, and the values of normalized maximum lactate (Lac_max_/Pyr_max_) compared. Alternatively the area under the curve (AUC) for hyperpolarized lactate was normalized to the AUC for hyperpolarized pyruvate and to cell number (Lac_auc_/Pyr_auc_) and values compared.

### Intracellular pH determination

Intracellular pH was determined using the chemical shift difference (A) between the intracellular inorganic phosphate (P_i_ int) and phosphocreatine (PCr) peaks in the ^31^P MR spectrum obtained at the start of the study (see above) and using the Henderson-Hasselbalch equation (pH= 6.77 + log [(A-3.29)/(5.68-A)] to calculate the pH as described previously [50].

### LDH activity assay

LDH activity was monitored using a spectrophotometric assay designed to monitor the rate of NADH consumption at 340 nm during LDH-catalyzed conversion of pyruvate to lactate in cell lysates as described previously [49, 51]. The K_m_ and V_max_ values were calculated by fitting the initial rate of NADH consumption to a Lineweaver–Burke plot.

### Western blotting

Cells (~10^7^) were lysed by sonication in cell lysis buffer (Cell signaling). Total cellular protein (~20 μg) was separated by sodium dodecyl sulphate polyacrylamide gel electrophoresis and transferred onto Immobilon-FL PVDF membrane (Millipore). Immunoblotting was performed using primary antibodies against MCT1 and MCT4 (Santa Cruz Biotechnology), LDHA and LDHB (Abcam), β-tubulin and Actin (Cell Signaling) followed by horseradish peroxidase-conjugated secondary antibodies (Cell Signaling). Images were developed onto autoradiographic film using an enhanced chemiluminescence substrate kit (Thermo Scientific) and quantified using ImageJ software (NIH).

### NAD^+^/NADH assay

Total cellular NADH concentration and the NAD^+^/NADH ratio were determined spectrophotometrically using a commercially available kit (Biovision) according to the manufacturer's instructions.

### DNA methylation analysis

Genomic DNA was isolated from the NHA parental line [43], NHAIDHwt or NHAIDHmut cells (n=2 each) and digested with 1 mg/ml proteinase K in lysis buffer (50 mM Tris-HCl, 1 mM EDTA, 0.5% SDS, pH 8) overnight at 55°C. After RNase treatment, DNA was phenol/chloroform extracted, precipitated with ethanol and resuspended in buffer containing 10 mM Tris-HCl, 1 mM EDTA, pH 8. DNA was bisulfite converted using the EZ DNA Methylation Kit (Zymo Research) and processed on Infinium HumanMethylation450 bead arrays (Illumina) according to the manufacturer's protocol. Probe-level signals for individual CpG sites were subject to both background and global dye-bias correction [52]. Probes that map to regions with known germline polymorphisms, to multiple genomic loci, or to either sex chromosome were filtered out [53]. The Limma (moderated t-test) approach was used to identify probes that were significantly different between NHAIDHwt and NHAIDHmut cells. Beta (β) values (the ratio between methylated probe intensity and total probe intensities, which can be interpreted as the percentage of methylation) were then calculated. To match the assumption of normality, the β values for NHAIDHwt and NHAIDHmut samples were transformed by the logit function and rescaled to the parental cell line scale. Each value was represented in terms of parental standard deviations (using Limma's inflated variance estimates) away from the parental mean. Finally, Δβ values (difference in β value between NHAIDHwt and NHAIDHmut cells) and p-values were assigned to the most significant probe difference within a gene.

### TCGA analysis

Patient biopsy data for diffuse low-grade gliomas (grade II and III) deposited in the TCGA database (http://cancergenome.nih.gov) was downloaded through the CBio Portal (http://www.cbioportal.org) [54, 55]. Mean normalized z-scores for mRNA levels were determined for mutant and wild-type IDH1 glioma datasets. Statistical significance of differences was assessed using a two-tailed Mann-Whitney test with p<0.05 considered significant.

### Statistical analysis

Unless otherwise stated, results are expressed as mean ± standard deviation (n≥3 unless otherwise specified) and statistical significance evaluated using a two-tailed Student's t-test assuming unequal variance with p<0.05 considered significant.

## SUPPLEMENTARY FIGURES AND TABLES


