# Supplementary material for: Mutant IDH1 expression is associated with down-regulation of monocarboxylate transporters
Source: Oncotarget. 2016 Apr 26;7(23):34942–55. doi: 10.18632/oncotarget.9006 (PMC5085201; doi:10.18632/oncotarget.9006)
Supplement: Supplementary file 1 [file oncotarget-07-34942-s001.pdf]

SUPPLEMENTARY FIGURE

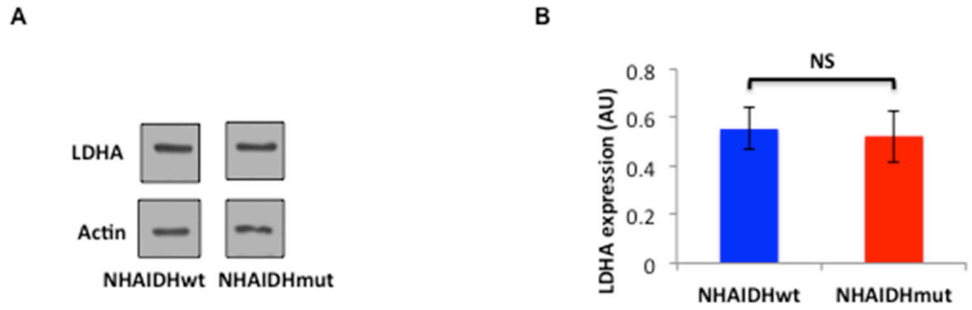

Supplementary Figure S1: A. Western blots for LDHA in NHAIDHwt and NHAIDHmut cells. **B.** Quantification of LDHA expression in NHAIDHwt and NHAIDHmut cells. NS indicates no significant difference.
